# Sex Hormone-Binding Globulin Prevents Carbon Tetrachloride-Induced Liver Fibrosis Development

**DOI:** 10.3390/ijms27114893

**Published:** 2026-05-28

**Authors:** Anna Álvarez-Guaita, Laura Briansó-Llort, Julia Cabrera-Serra, Lidia Fuertes-Rioja, Lorena Ramos-Pérez, María Teresa Salcedo-Allende, Cristina Hernández, Rafael Simó, David M. Selva

**Affiliations:** 1 Diabetes and Metabolism Research Unit, Vall d’Hebron Research Institute (VHIR), Vall d’Hebron University Hospital, 08035 Barcelona, Spain; anna.alvarezguaita@gmail.com (A.Á.-G.); laurabrianso@gmail.com (L.B.-L.); lidia.fuertes@vhir.org (L.F.-R.); lorena.ramos@vhir.org (L.R.-P.); cristina.hernandez@vhir.org (C.H.); rafael.simo@vhir.org (R.S.); 2 Autonomous University of Barcelona, 08193 Barcelona, Spain; 3 Diabetes and Metabolism Research Unit and CIBERDEM, Vall d’Hebron Research Institute (VHIR), 08035 Barcelona, Spain; 4 Human Pathology Department, Vall d’Hebron University Hospital, 08035 Barcelona, Spain; mteresa.salcedo@vallhebron.cat

**Keywords:** TGF-β1, metabolic dysfunction-associated steatohepatitis (MASH), sexual dimorphism collagen, liver, hepatic fibrosis, transgenic mice, metalloproteinases

## Abstract

Circulating sex hormone-binding globulin (SHBG) concentrations are lower in individuals with metabolic dysfunction-associated steatotic liver disease (MASLD) and metabolic dysfunction-associated steatohepatitis (MASH), reflecting its potential role in metabolic liver dysfunction. Our prior studies demonstrated that SHBG can attenuate MASLD by limiting hepatic lipid deposition, partly through suppression of lipogenic pathways, in both cellular and animal models. In the present work, we have examined whether SHBG could protect against development of liver fibrosis. For this purpose, in vitro and in vivo studies were performed. In vitro, we used co-cultures of human hepatocellular carcinoma cell line (HepG2) and human hepatic stellate cell line (LX-2) cells transfected using an SHBG expression vector vs. vehicle and treated with transforming growth factor beta 1 (TGF-β1). For in vivo studies we used wild-type and human *SHBG* transgenic mice developing liver fibrosis induced by carbon tetrachloride (CCl_4_). Our results clearly showed that SHBG overexpression reduced the TGF-β1-induced expression in collagen in LX-2 cells. Moreover, SHBG overexpression reduced the CCl_4_ induced liver fibrosis in both male and female mice. Histological examination revealed that *SHBG* transgenic mice had reduced NAS score and decreased collagen accumulation, assessed by Sirious Red staining. In addition, human *SHBG* transgenic mice treated with CCl_4_ exhibited lower collagen 1A1 (Col1A1) protein levels when compared with wild-type CCl_4_ treated mice. Mechanistically, SHBG attenuated fibrosis primarily through modulation of the TGF-β1/matrix metalloproteinases (MMPs)/tissue inhibitor metalloproteinases 1 (TIMP1) axis, characterized by reduced TGF-β1 levels, increased metalloprotease activity, and decreased TIMP1 levels compared with wild-type CCl_4_ treated mice. Notably, female *SHBG* transgenic mice exhibited greater protection against fibrosis than males, indicating a sex-dependent effect likely mediated by differences in sex steroid signaling. Taken together, we demonstrate for the first time that SHBG protects against liver fibrosis by promoting collagen degradation via the TGF-β1/MMPs/TIMP1 pathway. Further research is needed to elucidate the role of sex steroids in the regulation of MMPs and the observed sexual dimorphism.

## 1. Introduction

Sex hormone-binding globulin (SHBG) is a homodimeric glycoprotein primarily synthesized and secreted by the liver into the bloodstream, where it binds sex steroids with high affinity, thereby regulating their bioavailability and action [1,2]. Previous clinical studies have consistently reported reduced circulating SHBG levels in individuals with obesity, type 2 diabetes (T2D), and metabolic dysfunction-associated steatotic liver disease (MASLD) and metabolic dysfunction-associated steatohepatitis (MASH) [3,4,5,6]. Importantly, low circulating SHBG levels have also been associated with increased liver fibrosis severity in patients with metabolic liver disease, suggesting a potential link between SHBG deficiency and fibrogenesis [7,8]. In this regard, our previous work has uncovered several molecular mechanisms contributing to the downregulation of hepatic SHBG in these metabolic disorders. Specifically, we and others have shown that SHBG expression is suppressed by (a) hepatic lipid accumulation driven by high-carbohydrate diets [6], (b) elevated levels of pro-inflammatory cytokines such as tumor necrosis factor-alpha (TNF-α) and interleukin-1β (IL-1β) [9,10,11], and (c) increased hepatic levels of transforming growth factor-beta 1 (TGF-β1) [8], a known fibrogenic mediator [12,13,14,15].

Importantly, SHBG downregulation appears to contribute to disease pathogenesis. We have previously shown that in human *SHBG* transgenic mice, hepatic steatosis is mitigated in MASLD models, accompanied by decreased activity of key lipogenic enzymes such as ATP-citrate lyase (ACLY), acetyl-CoA carboxylase (ACC), and fatty acid synthase (FAS), as well as the transcription factor peroxisome proliferator-activated receptor gamma (PPARγ) [5]. These findings suggest that reduced SHBG levels in obesity and T2D may promote hepatic lipogenesis, thus facilitating the progression of MASLD. Together, these observations raise the possibility that SHBG may also play a role in later stages of disease progression, including fibrosis.

Given the regulatory role of SHBG in liver metabolism and inflammation, we hypothesized that SHBG attenuates liver fibrosis by inhibiting TGF-β1 signaling and restoring the balance between MMPs and TIMPs, thereby promoting extracellular matrix (ECM) degradation [16,17]. To study fibrosis in vivo, we used the CCl_4_-induced model in wild-type and *SHBG* transgenic mice, as rodents typically do not develop spontaneous liver fibrosis [18,19]. TGF-β1 plays a central role in CCl_4_-induced liver injury, promoting hepatic stellate cell activation and extracellular matrix accumulation, marking a central pathway in the transition from MASH to fibrosis [13,14,20].

In a healthy liver, the ECM is dynamically regulated by matrix metalloproteinases (MMPs) and their tissue inhibitors (TIMPs) and a critical balance between MMPs and TIMPs governs ECM remodeling [21,22]. During chronic liver injury, this balance is disrupted—particularly through the upregulation of TIMP-1, which inhibits MMP activity and limits collagen degradation, promoting fibrotic progression [21,22]. MMPs such as MMP-1, MMP-8, and MMP-13, also known as collagenases, are key enzymes that target fibrillar collagens (types I, II, III), and play a central role in ECM turnover [23].

To address this hypothesis, we performed complementary in vitro and in vivo experiments. In vitro, we aimed to determine whether SHBG modulates TGF-β1-induced pro-fibrotic responses in hepatic stellate cells [23]. In vivo, we aimed to evaluate whether SHBG overexpression protects against CCl_4_-induced liver fibrosis and to elucidate the underlying molecular mechanisms [24]. In this study, we showed for the first time that overexpression of SHBG protects against CCl_4_-induced liver fibrosis. Mechanistically, human *SHBG* transgenic mice exhibited reduced collagen deposition and TGF-β1 protein levels together with an enhanced MMP activity, suggesting a shift toward ECM degradation. These findings position SHBG as a potential therapeutic target for preventing or treating liver fibrosis in metabolic liver disease.

## 2. Results

### 2.1. SHBG Overexpression Reduced TGF-β1-Induced Increase in Collagen Expression in LX-2 Cells and HepG2 Cell Co-Cultures

**Figure 1:**
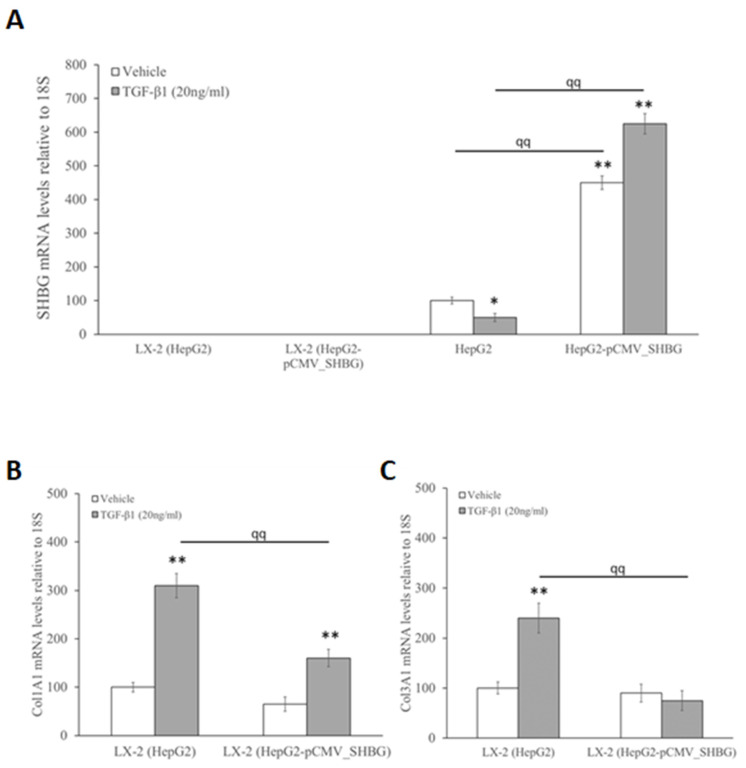
SHBG expression modulates TGF-β1-induced collagen gene expression in hepatic and hepatic stellate cell models. (**A**) SHBG mRNA expression levels were measured by qPCR in LX-2 cells co-cultured with HepG2 or HepG2 cells overexpressing SHBG (HepG2-pCMV_SHBG), and in HepG2 or HepG2-pCMV_SHBG cells treated with either vehicle or TGF-β1 (20 ng/mL) for 24 h. The SHBG expression is shown relative to 18S levels. (**B**) Expression analysis of collagen type I alpha 1 (Col1A1) mRNA levels in LX-2 cells co-cultured with HepG2 or HepG2-pCMV_SHBG cells, treated as in (**A**). (**C**) Expression analysis of collagen type III alpha 1 (Col3A1) mRNA levels in the same conditions as in (**A**). All data represent mean ± SEM from at least three independent experiments. Statistical significance determined by two-tailed Student’s *t*-test. * *p* < 0.05, ** *p* < 0.01 vs. vehicle-treated controls or as indicated. ^qq^ *p* < 0.01 when comparing HepG2 vs. pCMV-SHBG.

To investigate the role of SHBG in modulating collagen gene expression in hepatic stellate cells (HSCs), we established co-cultures of LX-2 cells with HepG2 or SHBG-overexpressing HepG2 cells (HepG2-pCMV_SHBG), and treated them with TGF-β1 (20 ng/mL), to induce LX-2 activation and to induce collagen expression as described previously [8,25], or vehicle for 24 h. The results showed that SHBG mRNA expression was undetectable in LX-2 cells alone in any condition, as expected (Figure 1A). In contrast, SHBG mRNA expression was detected in HepG2 cells and HepG2-pCMV_SHBG with significantly higher expression levels in HepG2-pCMV_SHBG when compared with HepG2 cells (Figure 1A). The SHBG expression was reduced in HepG2 cells when treated with TGF-β1 (20 ng/mL), while being increased in HepG2-pCMV_SHBG (Figure 1A).

We next assessed the expression of Col1A1 mRNA levels. The results showed that they were significantly induced by TGF-β1 in LX-2 cells co-cultured with HepG2 cells (~3-fold increase vs. vehicle) (Figure 1B). However, this TGF-β1-induced increase was markedly blunted in LX-2 cells co-cultured with HepG2-pCMV_SHBG (Figure 1B). Similarly, Col3A1 mRNA was elevated following TGF-β1 treatment in LX-2/HepG2 co-cultures, but not in co-cultures with SHBG-overexpressing HepG2 cells (Figure 1C).

### 2.2. SHBG Protected Against Liver Fibrosis Induced by CCl_4_

**Figure 2:**
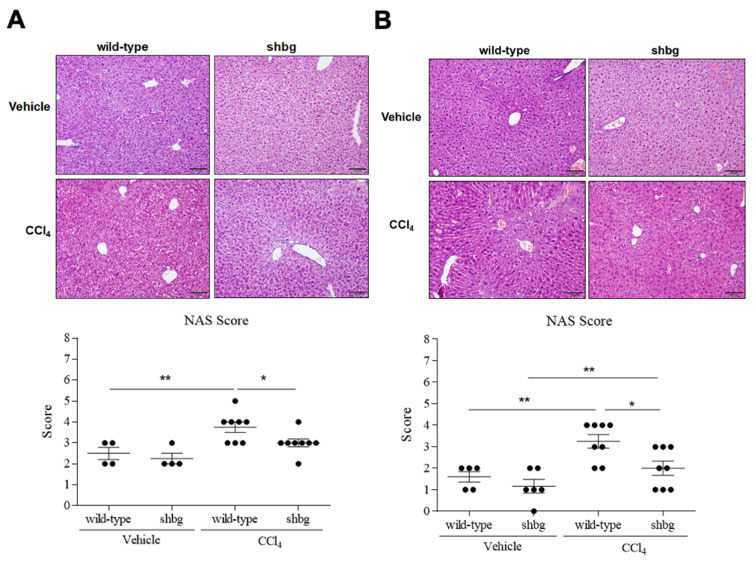
Liver H&E analysis of WT and human *SHBG* transgenic (SHBG) mice treated with vehicle or CCl_4_. (**A**) Representative H&E-stained liver sections (**top**) and quantification of NAS scores (**bottom**) in wild-type and SHBG female mice treated with vehicle or CCl_4_. (**B**) Representative H&E-stained liver sections (**top**) and quantification of NAS scores (**bottom**) in wild-type and SHBG female mice treated with vehicle or CCl_4_. In the bottom panels each dot represents an individual mouse. Data are presented as mean ± SEM. Scale bars in histology panels = 100 µm. Statistical significance determined by one-way ANOVA followed by post hoc testing. * *p* < 0.05, ** *p* < 0.01.

**Figure 3:**
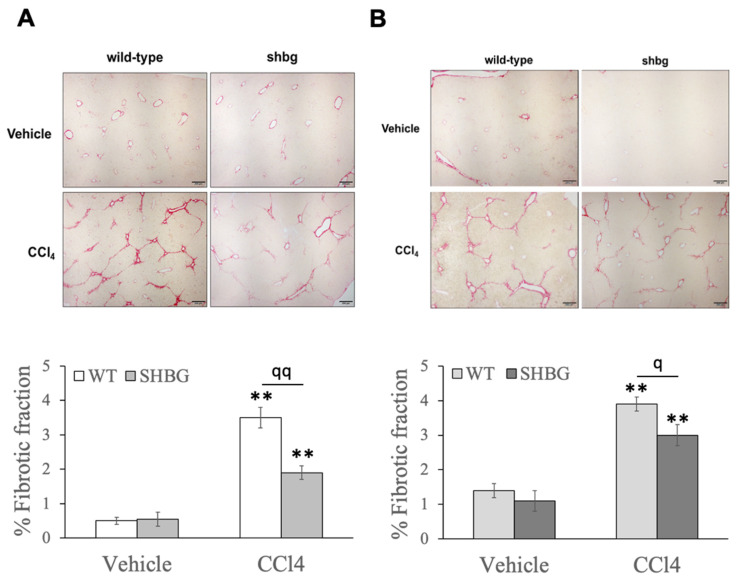
Liver picrosirius red staining analysis of WT and human *SHBG* transgenic (SHBG) mice treated with vehicle or CCl_4_. (**A**) Representative picrosirius red-stained liver sections (**top**) and quantification of fibrotic fraction (**bottom**) in wild-type and SHBG female mice treated with vehicle or CCl_4_. (**B**) Representative picrosirius red-stained liver sections (**top**) and quantification of fibrotic fraction (**bottom**) in wild-type and SHBG male mice treated with vehicle or CCl_4_. Data are presented as mean ± SEM. Scale bars in histology panels = 100 µm. Statistical significance determined by one-way ANOVA followed by post hoc testing ** *p* < 0.01 when comparing vehicle vs. CCl_4_. ^q^ *p* < 0.05, ^qq^ *p* < 0.01 when comparing WT vs. SHBG.

Wild-type (WT) and human *SHBG* transgenic (SHBG) mice were treated with vehicle or CCl_4_ twice a week for four weeks. After the treatment, H&E analysis by an independent pathologist revealed that both male and female WT mice treated with CCl_4_ had significantly increased NAS score when compared with WT and SHBG mice treated with vehicle (Figure 2A,B). The SHBG mice treated with CCl_4_ showed a significantly reduced NAS score when compared with WT mice treated with CCl_4_ (Figure 2A,B). Moreover, WT mice treated with CCl_4_ showed a clear and significant increase in liver fibrosis when compared with WT and SHBG vehicle-treated mice, as evidenced in histological sections stained with picrosirius red and the quantification of collagen deposition using the ImageJ software (Figure 3A,B). Remarkably, the SHBG mice treated with CCl_4_ had a significant reduction in the liver collagen deposition when compared with WT-treated CCl_4_ mice (Figure 3A,B).

### 2.3. SHBG Reduced Collagen and TGF-β1 Protein Levels in Liver Fibrosis Induced by CCl_4_

**Figure 4:**
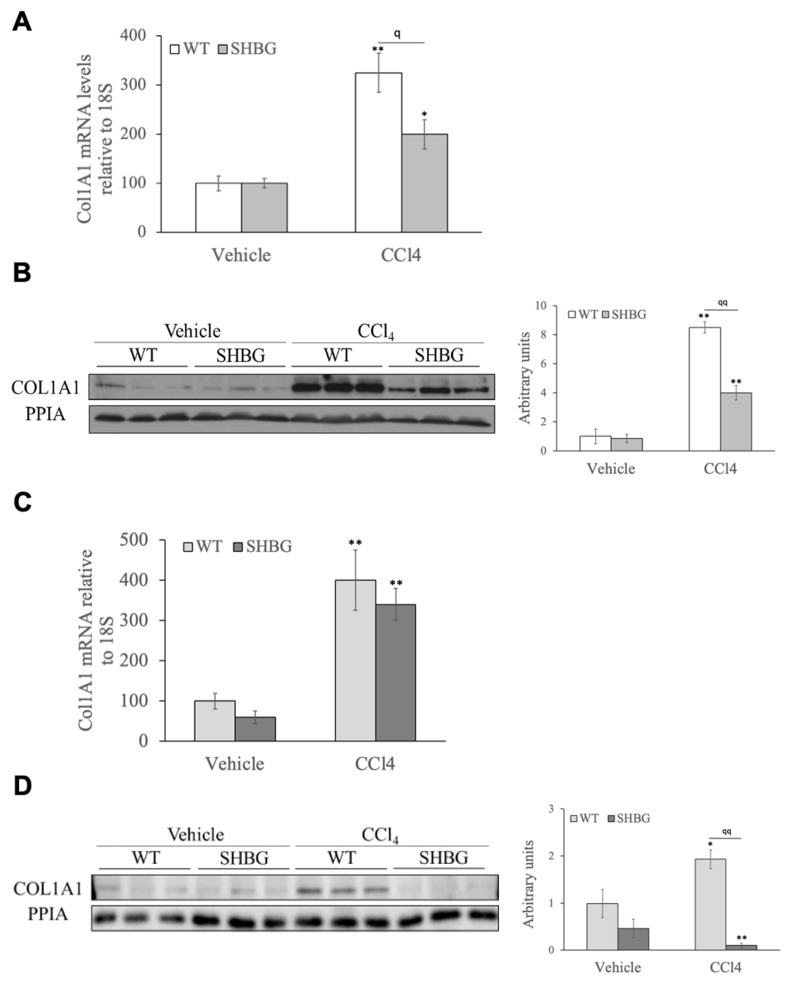
Hepatic Col1A1 mRNA and protein levels in WT and human *SHBG* transgenic (SHBG) mice treated with vehicle or CCl_4_. (**A**) Liver Col1A1 mRNA levels were determined in relation to 18S RNA in wild-type and SHBG female mice treated with vehicle or CCl_4_. Data points are mean ± SEM. (**B**) Liver Col1A1 protein levels were measured by Western blotting using PPIA as a housekeeping reference protein in wild-type and SHBG female mice treated with vehicle or CCl_4_ (*n* = 3 each). (**C**) Liver Col1A1 mRNA levels were determined in relation to 18S RNA in wild-type and SHBG male mice treated with vehicle or CCl_4_. Data points are mean ± SEM. (**D**) Liver Col1A1 protein levels were measured by Western blotting using PPIA as a housekeeping reference protein in wild-type and SHBG male mice treated with vehicle or CCl_4_ (*n* = 3 each). Statistical significance was accepted at * *p* < 0.05 and ** *p* < 0.01 when comparing vehicle vs. CCl_4_. ^q^ *p* < 0.05, ^qq^ *p* < 0.01 when comparing WT vs. SHBG.

We first analyzed the collagen mRNA expression in vehicle- and CCl_4_-treated WT and SHBG female mice. As expected, we found a significant increase in collagen expression in WT female mice treated with CCl_4_ when compared with WT and SHBG female vehicle-treated mice (Figure 4A). The SHBG CCl_4_-treated female mice showed a significant reduction in collagen mRNA levels when compared with WT CCl_4_-treated mice (Figure 4A). These results were confirmed at the protein level, since WT females treated with CCl_4_ showed a significant increase in collagen protein levels when compared with WT and SHBG female vehicle-treated mice (Figure 4B). Remarkably, SHBG CCl_4_-treated female mice showed a significant reduction in collagen protein levels when compared with WT CCl_4_-treated mice (Figure 4B).

We next analyzed the collagen mRNA expression in vehicle- and CCl_4_-treated WT and SHBG male mice. The results showed a significant increase in collagen expression in WT and SHBG male mice treated with CCl_4_ when compared with WT and SHBG female vehicle-treated mice (Figure 4A). There were no significant differences in collagen mRNA levels between WT and SHBG mice treated with CCl_4_ (Figure 4A). At a protein level, WT male mice treated with CCl_4_ showed a significant increase in collagen protein levels when compared with WT and SHBG male vehicle-treated mice (Figure 4B). The SHBG CCl_4_-treated male mice showed a significant reduction in collagen protein levels when compared with WT CCl_4_-treated mice (Figure 4B).

**Figure 5:**
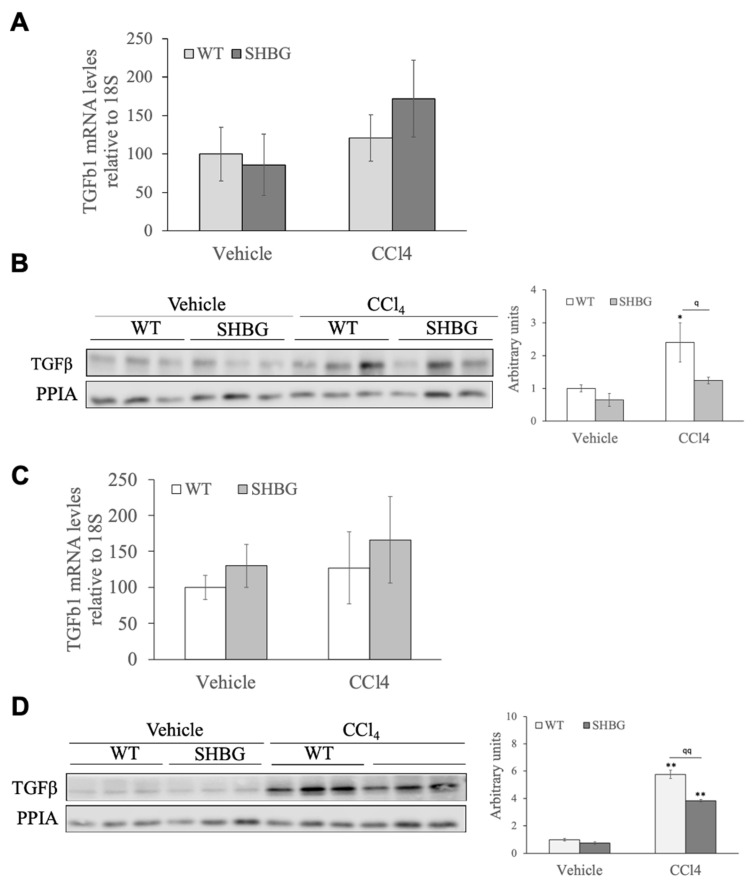
Hepatic TGF-β1 mRNA and protein levels in WT and human *SHBG* transgenic (SHBG) mice treated with vehicle or CCl_4_. (**A**) Liver TGF-β1 mRNA levels were determined in relation to 18S RNA in wild-type and SHBG female mice treated with vehicle or CCl_4_. Data points are mean ± SEM. (**B**) Liver TGF-β1 protein levels were measured by Western blotting using PPIA as a housekeeping reference protein in wild-type and SHBG female mice treated with vehicle or CCl_4_ (*n* = 3 each). (**C**) Liver TGF-β1 mRNA levels were determined in relation to 18S RNA in wild-type and SHBG male mice treated with vehicle or CCl_4_. Data points are mean ± SEM. (**D**) Liver TGF-β1 protein levels were measured by Western blotting using PPIA as a housekeeping reference protein in wild-type and SHBG male mice treated with vehicle or CCl_4_ (*n* = 3 each). Statistical significance was accepted at * *p* < 0.05 and ** *p* < 0.01 when comparing vehicle vs. CCl_4_. ^q^ *p* < 0.05, ^qq^ *p* < 0.01 when comparing WT vs. SHBG.

Since TGF-β1 plays a key role in fibrosis development [13], we decided to measure TGF-β1 mRNA and protein levels in the liver. The results showed that there were no significant differences in TGF-β1 mRNA levels between WT and SHBG female and male mice treated with vehicle or CCl4 (Figure 5A,C). However, at the protein level we observed a significant increase in TGF-β1 in WT female CCl_4_-treated mice when compared with WT and SHBG vehicle-treated mice (Figure 5B). The SHBG CCl_4_-treated female mice showed a significant reduction in TGF-β1 protein levels when compared with WT CCl_4_-treated mice (Figure 5B). Regarding male mice, the results showed a significant increase in TGF-β1 protein levels in WT and SHBG female CCl_4_-treated mice when compared with WT and SHBG vehicle-treated mice (Figure 5D). However, the SHBG CCl_4_-treated female mice showed a significant reduction in TGF-β1 protein levels when compared with WT CCl_4_-treated mice (Figure 5D).

### 2.4. SHBG Protection Against Liver Fibrosis Induced by CCl_4_ Is Related to an Increase in Metalloprotease Protein Levels

**Figure 6:**
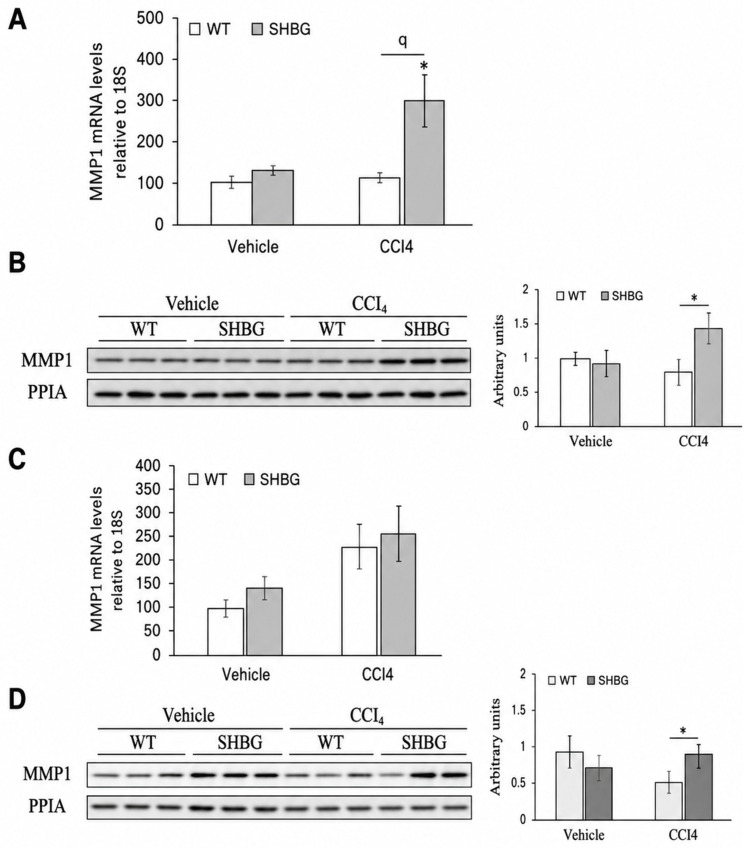
Hepatic MMP1 mRNA and protein levels in WT and human *SHBG* transgenic (SHBG) mice treated with vehicle or CCl_4_**.** (**A**) Liver MMP1 mRNA levels were determined in relation to 18S RNA in wild-type and SHBG female mice treated with vehicle or CCl_4_. Data points are mean ± SEM. (**B**) Liver MMP1 protein levels were measured by Western blotting using PPIA as a housekeeping reference protein in wild-type and SHBG female mice treated with vehicle or CCl_4_ (*n* = 3 each). (**C**) Liver MMP1 mRNA levels were determined in relation to 18S RNA in wild-type and SHBG male mice treated with vehicle or CCl_4_. Data points are mean ± SEM. (**D**) Liver MMP1 protein levels were measured by Western blotting using PPIA as a housekeeping reference protein in wild-type and SHBG male mice treated with vehicle or CCl_4_ (*n* = 3 each). Statistical significance was accepted at * *p* < 0.05 when comparing vehicle vs. CCl_4_. ^q^ *p* < 0.05, when comparing WT vs. SHBG.

**Figure 7:**
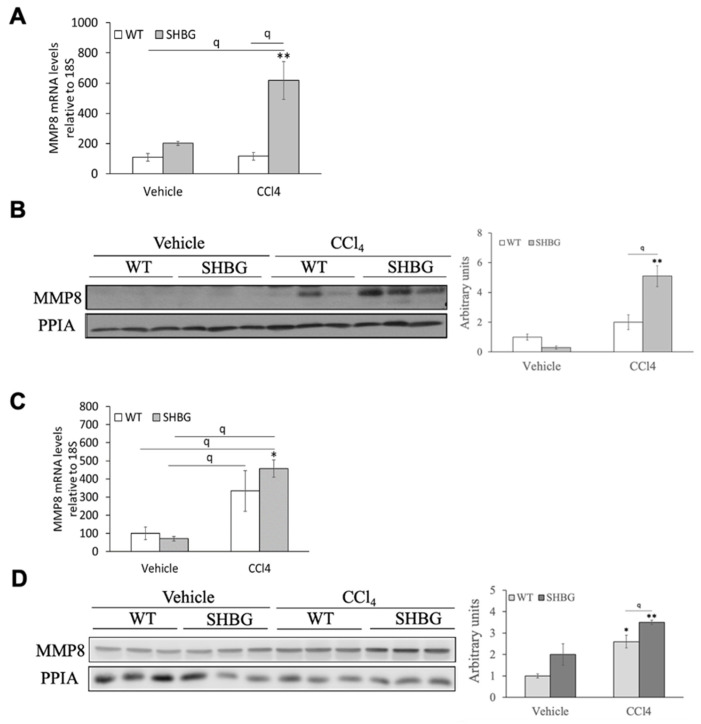
Hepatic MMP8 mRNA and protein levels in WT and human *SHBG* transgenic (SHBG) mice treated with vehicle or CCl_4_. (**A**) Liver MMP8 mRNA levels were determined in relation to 18S RNA in wild-type and SHBG female mice treated with vehicle or CCl_4_. Data points are mean ± SEM. (**B**) Liver MMP8 protein levels were measured by Western blotting using PPIA as a housekeeping reference protein in wild-type and SHBG female mice treated with vehicle or CCl_4_ (*n* = 3 each). (**C**) Liver MMP8 mRNA levels were determined in relation to 18S RNA in wild-type and SHBG male mice treated with vehicle or CCl_4_. Data points are mean ± SEM. (**D**) Liver MMP8 protein levels were measured by Western blotting using PPIA as a housekeeping reference protein in wild-type and SHBG male mice treated with vehicle or CCl_4_ (*n* = 3 each). Statistical significance was accepted at * *p* < 0.05 and ** *p* < 0.01 when comparing vehicle vs. CCl_4_. ^q^ *p* < 0.05 when comparing WT vs. SHBG.

**Figure 8:**
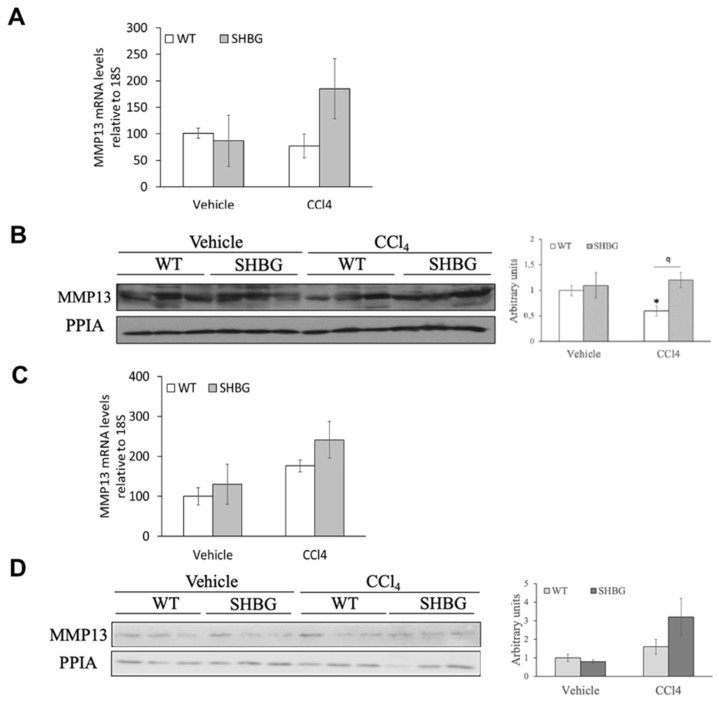
Hepatic MMP13 mRNA and protein levels in WT and human *SHBG* transgenic (SHBG) mice treated with vehicle or CCl_4_. (**A**) Liver MMP13 mRNA levels were determined in relation to 18S RNA in wild-type and SHBG female mice treated with vehicle or CCl_4_. Data points are mean ± SEM. (**B**) Liver MMP13 protein levels were measured by Western blotting using PPIA as a housekeeping reference protein in wild-type and SHBG female mice treated with vehicle or CCl_4_ (*n* = 3 each). (**C**) Liver MMP13 mRNA levels were determined in relation to 18S RNA in wild-type and SHBG male mice treated with vehicle or CCl_4_. Data points are mean ± SEM. (**D**) Liver MMP13 protein levels were measured by Western blotting using PPIA as a housekeeping reference protein in wild-type and SHBG male mice treated with vehicle or CCl_4_ (*n* = 3 each). Statistical significance was accepted at * *p* < 0.05 when comparing vehicle vs. CCl_4_. ^q^ *p* < 0.05 when comparing WT vs. SHBG.

**Figure 9:**
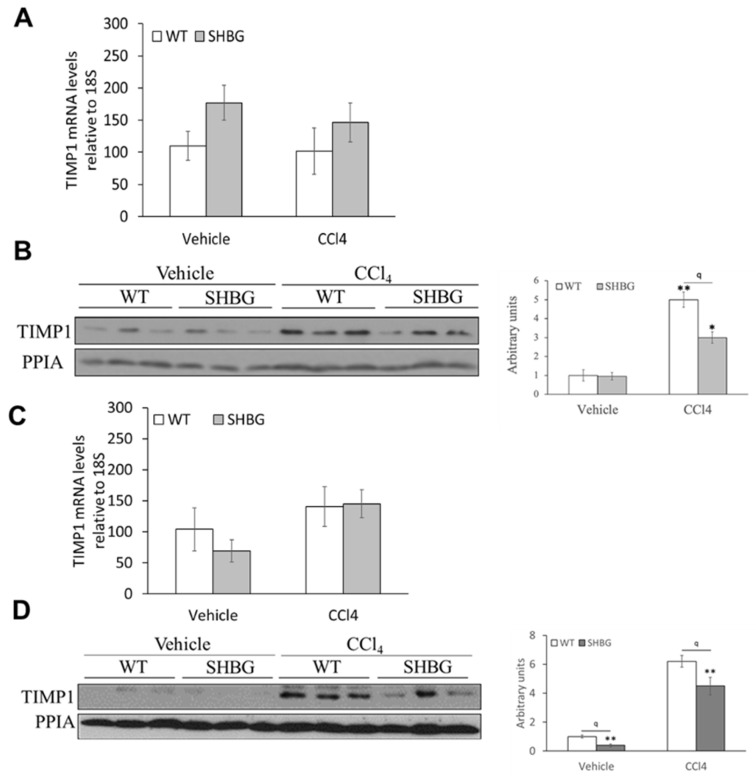
Hepatic TIMP1 mRNA and protein levels in WT and human *SHBG* transgenic (SHBG) mice treated with vehicle or CCl_4_. (**A**) Liver TIMP1 mRNA levels were determined in relation to 18S RNA in wild-type and SHBG female mice treated with vehicle or CCl_4_. Data points are mean ± SEM. (**B**) Liver TIMP1 protein levels were measured by Western blotting using PPIA as a housekeeping reference protein in wild-type and SHBG female mice treated with vehicle or CCl_4_ (*n* = 3 each). (**C**) Liver TIMP1 mRNA levels were determined in relation to 18S RNA in wild-type and SHBG male mice treated with vehicle or CCl_4_. Data points are mean ± SEM. (**D**) Liver TIMP1 protein levels were measured by Western blotting using PPIA as a housekeeping reference protein in wild-type and SHBG male mice treated with vehicle or CCl_4_ (*n* = 3 each). Statistical significance was accepted at * *p* < 0.05 and ** *p* < 0.01 when comparing vehicle vs. CCl_4_. ^q^ *p* < 0.05 when comparing WT vs. SHBG.

To unravel the underlying mechanisms involved in SHBG capacity of reducing collagen accumulation after CCl_4_ treatment, we decided to determine the mRNA and protein levels of MMP1, MMP8 and MMP13, three well-known MMPs that degrade collagen. In female mice, the results showed that SHBG mice treated with CCl_4_ had significantly higher mRNA and protein levels of MMP1 and MMP8 and higher protein levels of MMP13 when compared with WT treated with CCl_4_ (Figure 6A,B, Figure 7A,B and Figure 8B). This was accompanied by a significant reduction in TIMP1 protein levels in SHBG females treated with CCl_4_ when compared with WT treated with CCl_4_ (Figure 9B). In male mice, the results showed no differences in mRNA levels of MMP1, MMP8, MMP13 and TIMP1 between WT and SHBG mice treated with CCl_4_ (Figure 6C, Figure 7C, Figure 8C and Figure 9C). Moreover, SHBG mice treated with CCl_4_ had significantly higher protein levels of MMP1 and MMP8 when compared with WT treated with CCl_4_ (Figure 6D and Figure 7D) but no significant differences were found in MMP13 protein levels (Figure 8D). This was accompanied by a significant reduction in TIMP1 protein levels in SHBG mice treated with CCl_4_ when compared with WT treated with CCl_4_ (Figure 9D).

## 3. Discussion

This study shows that human SHBG overexpression protects against CCl_4_-induced liver fibrosis, with more pronounced effects in female mice than in males. The sexual dimorphism found in our study aligns with evidence reported in the literature describing the sex-specific regulation in fibrosis development and progression [26,27]. In both sexes, human *SHBG* transgenic mice treated with CCl_4_ showed reduced NAS scores and diminished collagen deposition when compared to wild-type littermate vehicle-treated controls. Therefore, our results provide new insights into the role of SHBG in liver pathophysiology and identify SHBG as a potential therapeutic target in liver fibrosis.

Reduced SHBG levels have been linked to obesity, type 2 diabetes, and metabolic liver diseases, conditions that commonly coincide with hepatic lipid accumulation and fibrosis progression [28,29,30]. Using in vitro and in vivo approaches, we have previously described the molecular mechanisms by which hepatic SHBG expression is downregulated in MASLD and MASH [6,8,31]. Importantly, we have reported that human *SHBG* transgenic mice were protected against lipid accumulation in the liver using two different mouse models of MASLD [5]. Our findings were corroborated in a meta-analysis performed by Jaruvongvanich et al., who concluded that higher SHBG levels were associated with lower MASLD odds in both men and women [32]. With this evidence, in our present work we wanted to investigate whether SHBG may also modulate liver fibrosis, a later stage in liver disease.

To investigate a possible role of SHBG in regulating fibrogenesis, we first decided to use an in vitro approach consisting of a co-culture model using LX-2 cells and HepG2 cells. Our data showed that overexpression of SHBG in HepG2 cells significantly attenuated TGF-β1-induced increase in Col1A1 and Col3A1 mRNA expression in LX-2 cells, suggesting that SHBG exerted anti-fibrotic paracrine effects on HSCs. These findings suggest that, although SHBG is classically recognized as a circulating sex steroid-binding protein, its anti-fibrotic effects appear to be independent of sex steroid modulation and may involve a more direct action on hepatic stellate cells. Overall, our data support a model of hepatocyte–stellate cell crosstalk, in which SHBG produced by hepatocytes exerts direct regulatory effects on hepatic stellate cells, beyond its canonical endocrine function. Notably, SHBG mRNA expression was significantly downregulated in HepG2 cells in the presence of TGF-β1, as previously shown [8], while increased in HepG2-pCMV_SHBG cells under the same conditions. Since the pCMV promoter is not responsive to TGF-β1, our findings support the hypothesis that SHBG may counteract TGF-β1-induced pro-fibrogenic signaling. Mechanistically, SHBG may interfere with pro-fibrogenic signaling by attenuating TGF-β1 downstream pathways, particularly by reducing phosphorylation and nuclear translocation of SMAD2/3, thereby limiting transcription of fibrogenic genes such as COL1A1 and COL3A1. This suggests that SHBG acts upstream of canonical SMAD signaling and ultimately restrains hepatic stellate cell activation.

To induce liver fibrosis in mice, we used carbon tetrachloride (CCl_4_). Repeated CCl_4_ injections trigger hepatocyte injury, oxidative stress, and inflammation, which activate hepatic stellate cells and drive collagen deposition, ultimately resulting in progressive fibrosis [13,20,33]. The CCl_4_-induced liver fibrosis model mimics several features of human liver fibrosis, such as: ECM accumulation and collagen type I expression, TGF-β1 upregulation and activation of HSCs, and TIMP/MMP imbalance [13,20,33]. After treating human *SHBG* transgenic mice and their WT littermates with vehicle and CCl_4_, we observed a marked reduction in NAS scores and Sirius Red-stained collagen deposition in *SHBG* transgenic mice compared with their WT mice. While reduced fibrosis was evident in both sexes, the effect was more pronounced in females, suggesting a potential sex-specific mechanism in SHBG-mediated protection. Our results agree with previous reports found in the literature describing that female rodents were shown to be more resistant to liver injury and fibrosis following CCl_4_ exposure, having lower collagen deposition [34,35,36]. The collagen reduction in human *SHBG* transgenic mice was also confirmed at the mRNA and protein levels in human *SHBG* female mice and at the protein level in human *SHBG* male mice. This observation is consistent with known sex-specific patterns in liver fibrosis and the protective role of estradiol in female rats treated with CCl_4_ and dimethylnitrosamine to induce liver fibrosis [36,37]. The stronger anti-fibrotic effect observed in females may be explained by estradiol-mediated reinforcement of anti-fibrotic signaling, which enhances repression of fibrogenic gene transcription and synergizes with SHBG-dependent inhibition of collagen production pathways. In contrast, the absence of this estrogenic reinforcement in males may explain why SHBG predominantly affects COL1A1 at the protein level without a comparable reduction in mRNA expression. Furthermore, in *SHBG* transgenic mice, hepatic TGF-β1 protein levels were decreased, suggesting that SHBG may modulate this key player in the pro-fibrogenic signaling pathway [13,14,15]. Notably, this reduction was observed only at a protein level, suggesting that SHBG may influence TGF-β1 post-transcriptionally, possibly via changes in secretion, degradation, or upstream inflammatory signals. In this regard, studies involving the determination of decorin and Smad7 will be performed in the near future. Given the central role of TGF-β1 in mediating the fibrotic transition in chronic liver injury, SHBG’s ability to downregulate this cytokine may represent a key anti-fibrotic mechanism. In this regard, a recent report published by Lee and collaborators showed that SHBG transgenic mice are protected from liver fibrosis under chronic liver inflammatory conditions in a model of liver fibrosis, cirrhosis and hepatocellular carcinoma induced by Diethylnitrosamine (DEN) and N-Nitrosomorpholine (NMOR) [38].

In addition to reducing collagen synthesis, SHBG enhanced collagen breakdown by upregulating hepatic MMPs (MMP1, MMP8, MMP13), enzymes known to degrade collagens [23], while simultaneously reducing TIMP1, a natural inhibitor of these enzymes [21,22]. The observed modulation of MMP and TIMP1 levels indicates that SHBG may help reestablish the disrupted MMP/TIMP equilibrium during fibrosis, promoting extracellular matrix remodeling and resolution. Interestingly, while both male and female *SHBG* transgenic mice showed increased MMP1 and MMP8 protein levels, only female mice demonstrated a significant increase in MMP13. This sex-specific regulation of MMP13 may be mechanistically relevant, particularly in females, and could reflect differential hormonal or signaling contexts that influence SHBG-mediated extracellular matrix remodeling. To the best of our knowledge, sex-specific regulation of hepatic MMPs has not been extensively studied, and warrants further research.

From a translational perspective, these results open the possibility of developing SHBG-based or SHBG-mimetic therapies aimed at halting or reversing hepatic fibrosis. Given the high prevalence of metabolic diseases associated with low SHBG levels, enhancing SHBG function may provide a dual therapeutic benefit, reducing both hepatic steatosis and fibrosis progression. The CCl_4_-induced fibrosis model, which reflects toxic injury-driven fibrosis and may not fully represent diet-induced MASLD/MASH-associated fibrosis, is a limitation of this study. In addition, the in vitro model using HepG2 cells instead of primary hepatocytes may not fully recapitulate physiological SHBG production and signaling, as well as the absence of other relevant liver cell types such as Kupffer cells or liver sinusoidal endothelial cells. Furthermore, SHBG overexpression in this setting is artificial and may not accurately reflect endogenous physiological conditions.

In conclusion, our study identifies SHBG as a novel regulator of liver fibrosis. Through suppression of TGF-β1 protein expression and promotion of collagen degradation via increased MMP activity, SHBG overexpression protects against CCl_4_-induced hepatic fibrosis; however, we found differences between sexes. These findings position SHBG as a promising target in the development of anti-fibrotic strategies, particularly in the context of metabolic liver disease.

## 4. Materials and Methods

### 4.1. Cell Culture Experiments

Cell culture reagents were purchased from Life Technologies Inc. (Invitrogen SA, Barcelona, Spain). HepG2 hepatoblastoma cells (cat. no. HB-8065; ATCC, LGC Standards SLU, Barcelona, Spain) were maintained in Dulbecco’s Modified Eagle Medium supplemented with 10% fetal bovine serum and antibiotics (100 U/mL penicillin and 100 μg/mL streptomycin), at 37 °C in a humidified atmosphere containing 5% CO_2_. HepG2 cells overexpressing SHBG were achieved by stable transfection using an SHBG expression vector (pCMV-SHBG) as described before [5]. An empty vector (pCMV) was used as a control. The pCMV and pCMV-SHBG vectors were kindly provided by Dr. Geoffrey Hammond, (University of British, Vancouver, BC, Canada). LX-2 cells (human hepatic stellate cell line, SCC064, Merck Life Science S.L.U, Spain) were maintained in Dulbecco’s Modified Eagle Medium (DMEM) supplemented with 2% fetal bovine serum (100 U/mL penicillin, and 100 µg/mL streptomycin) in the same conditions described above. LX-2 (RRID:CVCL_5792) and HepG2 (RRID:CVCL_0027) were authenticated by the supplier using short tandem repeat (STR) profiling (≥95–100% match with the reference STR profile) and were routinely tested for mycoplasma contamination. Cells were used for experiments within a limited number of passages after thawing according to the suppliers’ recommended protocols.

For co-culture experiments, HepG2 or pCMV_SHBG HepG2 cells were cultured in 6-well plates to 70−80% confluence in DMEM supplemented with 2% FBS and antibiotics. Transwells (Sarstedt, 833,930.041, Nümbrecht, Germany) containing LX-2 cells at 80% confluence were added for 24 h in the 6-well plates. Media from the wells and transwells were replaced by fresh DMEM containing 0.2% FBS and antibiotics. After 24 h, LX-2 cells were treated with vehicle or TGF-β1 (20 ng/mL) (Milteny Biotec, Madrid, Spain) for 24 h. After treatments, cells were scraped for RNA isolation. All experiments were performed in triplicate and in at least three different experiments.

### 4.2. Animals

Human *SHBG* transgenic mice and their wild-type littermates were used in this study. SHBG transgenic mice were on a C57BL/6 background. All mice used in this study were approximately 3 months of age. The body weights of males and females were 25–30 g and 20–25 g, respectively. The human *SHBG* transgenic mice contain a 4.3 kb region of the human shbg locus, which includes approximately 0.9 kb of the promoter region together with the coding region. These mice express human SHBG in their livers, which results in the presence of human SHBG in their blood [39]. Mice were maintained under standard conditions with food (Global Diet 2018; Harlan Interfauna Iberica, Barcelona, Spain) and water provided ad libitum and a 12 h light/dark cycle. Experimental procedures were approved by the Institutional Animal Use Subcommittees of Vall Hebron University Hospital Research Institute and the Universitat Autònoma de Barcelona (registry no. 45/13, Animal Experimental Ethics Committee).

### 4.3. In Vivo Experiments

Hepatic fibrosis was induced using carbon tetrachloride (CCl_4_) as described previously [15]. Briefly, mice received intraperitoneal (IP) injection of CCl_4_ (1 mL/kg) diluted in olive oil (1-part CCl_4_ and 3-parts olive oil, hence 4 mL/kg of total volume) or olive oil control (4 mL/kg). In an experiment lasting 4 weeks, male and female wild-type and human *SHBG* transgenic mice were divided randomly into two groups. The control group, female wild-type (*n* = 4), female SHBG (*n* = 4), male wild-type (*n* = 5) and male SHBG (*n* = 6) mice were given intraperitoneal (i.p.) injections of vehicle (olive oil) twice a week for the four weeks; and the treatment group, female wild-type (*n* = 8), female SHBG (*n* = 8), male wild-type (*n* = 8) and male SHBG (*n* = 8) mice were given i.p. injections of CCl_4_ twice a week for the four weeks. At the end of the experiment, livers were taken for histology, RNA and protein analysis. Animals were randomly assigned to experimental groups, and outcome assessments were performed by investigators blinded.

### 4.4. Histology

For morphological studies, livers were fixed in 4% paraformaldehyde for 24 h and embedded in paraffin. Serial 5-μm-thick sections were stained with H&E and picrosirius red for histological examination following standard procedures. Briefly, sections were alcohol–xylol-hydrated at room temperature, and subsequently, they were stained at room temperature in a solution of 0.1% Direct Red 80 (Sigma-Aldrich, Madrid, Spain) in saturated aqueous solution of picric acid (Sigma-Aldrich) for one hour. Following that, sections were rinsed twice in 0.5% acetic acid for 5 min, alcohol–xylol-dehydrated and covered with Eukitt mounting medium (Sigma-Aldrich). These sections were examined with an Olympus BX61 microscope. Six random fields from each slide were quantified using ImageJ software (ImageJ 1.x bundled with 64-bit Java 8) to determine the liver collagen deposition as the red-stained area in the image.

The stage of liver fibrosis (NAS score) was assessed blinded by an experienced pathologist according to the NASH-CRN scoring system. “The NASH-CRN system is based on three components: steatosis (0–3, reflecting the percentage of fat in hepatocytes), lobular inflammation (0–3, based on the number of inflammatory foci per field), and hepatocellular ballooning (0–2, indicating the degree of hepatocyte injury). These are summed to a total score of 0–8, where higher scores indicate greater disease activity” [40].

### 4.5. RNA Analysis

Total RNA was extracted from HepG2 and LX-2 cells, as well as mouse liver samples using TRIzol reagent (Invitrogen SA). Reverse transcription (RT) was performed at 42 °C for 50 min using 3 µg of total RNA and 200 U of Superscript II together with oligo-dT primers and reagents provided by Invitrogen. An aliquot of the RT product was amplified in a 20 µL reaction real-time PCR using SYBRGreen (Invitrogen SA) with appropriate oligonucleotide primer pairs corresponding to human *Col1A1, Col3A1* and *18S* and mouse *Col1a1, TGF-β1* and *18S* (Supplementary Table S1). The results were analyzed using the LightCycler^®^ 480 Software, Version 1.5.1 (Roche Diagnostics, Barcelona, Spain).

### 4.6. Western Blot Analysis

Mouse livers were homogenized in RIPA buffer with Complete protease inhibitor cocktail (Roche Diagnostics, Madrid, Spain). Protein extracts were used for Western blotting with antibodies against COL1A1, TGF-β1, MMP-1, MMP-8, MMP-13, TIMP1 and PPIA (Supplementary Table S2). Specific antibody–antigen complexes were identified using an HRP-labeled goat anti-rabbit IgG (P0448, Dako, Glostrup, Denmark), rabbit anti-mouse IgG (P0260, Dako), or rabbit anti-goat IgG (P0449, Dako), and Immobilon chemiluminescent HRP substrate (Merck-Millipore, Darmstadt, Germany) was used for detection by exposure to X-ray film.

### 4.7. Statistical Analyses

The normal distribution of the variables was evaluated using the Kolmogorov–Smirnov test. All quantitative variables followed a normal distribution and comparisons were performed using Student’s *t*-test for two groups, and one-way ANOVA with Bonferroni post hoc test for multiple groups. All data are presented as mean ± standard error of the mean. Significance was accepted at the level of * *p* < 0.05, ** *p <* 0.01 and *^q^ *p* < 0.05, **^qq^ *p* < 0.01. Statistical analyses were performed with the GraphPad Prism software (GraphPad 6.01 for Windows).

## Data Availability

Due to patentability issues, the data supporting the findings of this study are available from the corresponding author upon reasonable request.

## Supplementary Materials

The following supporting information can be downloaded at https://www.mdpi.com/article/10.3390/ijms27114893/s1.

## Abbreviations

CCl_4_	tetrachloride
COL1A1	collagen type I alpha 1 chains
SHBG	sex hormone-binding globulin
MMP	metalloproteinase
