# Supplementary material for: Sex Hormone-Binding Globulin Prevents Carbon Tetrachloride-Induced Liver Fibrosis Development
Source: Int J Mol Sci. 2026 May 28;27(11):4893. doi: 10.3390/ijms27114893 (PMC13256505; doi:10.3390/ijms27114893)
Supplement: Supplementary file 1 [file ijms-27-04893-s001.zip › ijms-4288750-supplementary.pdf]

Supplementary Table S1. Oligonucleotide primer pairs used in real-time PCR experiments.

Gene	Forward	Reverse
**Mouse *Col1a1***	GCTCCTCTTAGGGGCCACT	ATTGGGGACCCTTAGGCCAT
**Mouse *Tgfβ1***	AGGGCTACCATGCCAACTTC	CCACGTAGTAGACGATGGG
**Mouse *18s***	AGGGTTCGATTCCGGAGAGG	CAACTTTAATATACGCTATTGG
**Human Col1A1**	GAGGGCCAAGACGAAGACATC	CAGATCACGTCATCGCACAAC
**Human Col1A3**	GGAGCTGGCTACTTCTCGC	GGGAACATCCTCCTTCAACAG
**Human 18S**	TAACGAACGAGACTCTGGCAT	CGGACATCTAAGGGCATCACAG

Supplementary Table S2. Antibodies used for Western Blot.

Protein target	Manufacturer, catalog #	Species; monoclonal or polyclonal	Dilution used
COL1A1	BIOSS antibodies, USA (#BS-10423R)	Rabbit polyclonal	1:1000
TGF-β1	Abcam (ab215715)	Rabbit monoclonal	1:1000
MMP-1	Abcam (ab137322)	Rabbit polyclonal	1:1000
MMP-8	Santa Cruz (sc-514803)	Mouse monoclonal	1:1000
MMP-13	Abcam (ab315267)	Rabbit monoclonal	1:1000
TIMP1	R&D Systems (AF970)	Goat polyclonal	1:100
PPIA	Enzo Life Sciences, USA (#BML-SA296)	Rabbit monoclonal	1:20000
